# A Mixed-Methods Analysis of Mobile ACT Responses From Two Cohorts

**DOI:** 10.3389/fdgth.2022.869143

**Published:** 2022-05-12

**Authors:** Sydney Hoel, Amanda Victory, Tijana Sagorac Gruichich, Zachary N. Stowe, Melvin G. McInnis, Amy Cochran, Emily B. K. Thomas

**Affiliations:** ^1^Psychiatry, University of Wisconsin-Madison, Madison, WI, United States; ^2^Psychiatry, University of Michigan, Ann Arbor, MI, United States; ^3^Population Health Sciences and Mathematics, University of Wisconsin-Madison, Madison, WI, United States; ^4^Psychological and Brain Sciences, University of Iowa, Iowa City, IA, United States

**Keywords:** mobile health (mHealth), acceptance and commitment therapy (ACT), engagement, bipolar disorder, first-generation college students (FGCS), psychological flexibility, mixed methods, research methodology

## Abstract

**Background:**

Mobile transdiagnostic therapies offer a solution to the challenges of limited access to psychological care. However, it is unclear if individuals can actively synthesize and adopt concepts and skills via an app without clinician support.

**Aims:**

The present study measured comprehension of and engagement with a mobile acceptance and commitment therapy (ACT) intervention in two independent cohorts. Authors hypothesized that participants would recognize that behaviors can be flexible in form and function and respond in an ACT process-aligned manner.

**Methods:**

Mixed-methods analyses were performed on open-ended responses collected from initial participants (*n* = 49) in two parallel micro-randomized trials with: 1) first-generation college students (FGCSs) (*n* = 25) from a four-year public research university and 2) individuals diagnosed with bipolar disorder (BP) (*n* = 24). Twice each day over six weeks, participants responded to questions about mood and behavior, after which they had a 50-50 chance of receiving an ACT-based intervention. Participants identified current behavior and categorized behavior as values-based or avoidant. Interventions were selected randomly from 84 possible prompts, each targeting one ACT process: engagement with values, openness to internal experiences, or self-awareness. Participants were randomly assigned to either exploratory (10 FGCS, 9 BP) or confirmatory (15 FGCS, 15 BP) groups for analyses. Responses from the exploratory group were used to inductively derive a qualitative coding system. This system was used to code responses in the confirmatory group. Coded confirmatory data were used for final analyses.

**Results:**

Over 50% of participants in both cohorts submitted a non-blank response 100% of the time. For over 50% of participants, intervention responses aligned with the target ACT process for at least 96% of the time (FGCS) and 91% of the time (BP), and current behavior was labeled as values-based 70% (FGCS) and 85% (BP) of the time. Participants labeled similar behaviors flexibly as either values-based or avoidant in different contexts. Dominant themes were needs-based behaviors, interpersonal and family relationships, education, and time as a cost.

**Conclusions:**

Both cohorts were engaged with the app, as demonstrated by responses that aligned with ACT processes. This suggests that participants had some level of understanding that behavior can be flexible in form and function.

## Introduction

Given the increasing use of technology in daily life, mobile health apps offer solutions for filling in gaps in mental health care (1, 2). Mobile health apps contribute to the management of several mental health conditions, including bipolar disorder (BP) (3, 4), borderline personality disorder (5), major depressive disorder (6, 7), anxiety disorders (8), and posttraumatic stress disorder (9–11). In addition, they offer techniques to monitor internal thoughts and emotions outside of *in-person* care sessions and in the relevant moments of daily experience; personal awareness of such internal experiences is often a therapeutic goal. In the current mental health care model, such awareness is often retrospective and conveyed in the clinical sessions. Mobile health apps directly address many gaps in health systems, including clinician availability, constraints of transportation, health insurance, and cost, among others. For example, Tondo et al. reported that increasing access to care can improve even severe symptoms of depression such as suicidality (12). To build on these successes, we sought to investigate the quality of engagement with an intervention delivered in a mental health app without the support of a clinician to help navigate the intervention.

Realizing the promise of mental health apps requires addressing several barriers. The majority of mental health apps have yet to be evaluated for their efficacy (13, 14). Further, apps that target specific psychiatric diagnoses or operate around a specific treatment may not be useful to an individual who is unable to access care and obtain a diagnosis or treatment recommendation. There are groups who are at a high risk for experiencing psychological distress yet not characterized by a psychiatric diagnosis and may benefit from a mental health app. For example, numerous studies have identified college students as needing mental health care but not seeking it out (15, 16). Mental health apps may be a viable option for this population. Choosing among the multitude of apps available may be overwhelming and negatively impact engagement. For example, many apps allow users to monitor their symptoms over time. This can be helpful for some psychiatric diagnoses, but potentially detrimental to others without additional clinical support (17, 18). High attrition rates are common among mHealth interventions (19). Zucchelli et al. noted 53% of participants completed four out of six app-delivered 30-min ACT sessions in a study focused on alleviating psychosocial appearance concerns of those with atypical appearances via ACT (20). However, 60% of participants reported finding the interventions helpful, 88.6% said they were easy to understand, and exit interviews revealed daily reminders were important in encouraging app usage (20). Nevertheless, there is limited research on how best to characterize engagement among mHealth ACT interventions, and more work is needed to understand what predicts greater engagement with digital health interventions. Overcoming these issues may require mental health apps that provide additional clinical support beyond symptom monitoring, deliver interventions across diagnoses, and have been evaluated empirically.

A critical metric is the actual engagement with the process targeted by the app (e.g. monitoring or intervention). Clinicians will actively encourage treatment adherence and guide understanding of therapeutic processes. However, those who seek treatment through an app while not concurrently in the care of a clinician may not engage with the app in the manner expected to achieve the desired outcome. Measures of success include a) the success of the user experiencing the desired outcomes (i.e., symptom reduction, an increase in health-promoting behaviors), and b), successfully engaging users with the app itself. One would assume that desired outcomes are caused by high engagement, but this may not be the case, and if it is, the *degree* of engagement required to produce a positive effect may not be clear.

Acceptance and commitment therapy (ACT) is a transdiagnostic mindfulness-based therapy that targets experiential avoidance and encourages openness to internal experiences (e.g., emotions, thoughts), awareness of the function of behavior, and behavioral engagement with one's values (21). The driving principle of ACT is that pursuit of personally chosen values, vitality, and personal fulfillment are attainable, even when living with distressing experiences. ACT aims to increase psychological flexibility, the ability to engage in behavior that is consistent with one's values even when challenging or distressing (21). An important component of ACT is to reduce reliance on experiential avoidance, which is the inability or unwillingness to experience thoughts, emotions, physical sensations, or memories. Avoidance may provide relief in the moment, but in the long-term, it reduces contact with valued life directions and worsens the intensity and duration of the avoided stimulus. In contrast, psychological flexibility is associated with an increase in well-being and a reduction in symptoms (22).

ACT is effective in treating a variety of study populations (23), including cancer patients experiencing psychological symptoms (24) and substance abuse disorders (25, 26). In a study on nicotine addiction, Bricker et al. utilized the fundamental approach of ACT—to *accept* smoking triggers in adults attempting to quit rather than to avoid smoking triggers—when designing their smartphone application, iCanQuit (26). At the 12-month follow-up mark, the participants who used iCanQuit had 1.49 greater odds of quitting smoking when compared to a second group of participants who used an app called QuitGuide created by the National Cancer Institute, which focuses on avoidance (*p* < 0.001). Mobile ACT interventions have also been used concurrently with in-person ACT, as is the case with the ACT Daily app prototype, which was used with 14 patients with depression as they received treatment from an ACT clinician (27). In another study, a sample of college students showed improvement in depressive symptoms after completing an online, guided ACT intervention (28).

Further support for the efficacy of ACT when delivered virtually comes from positive outcomes when internet ACT, or iACT, is studied among individuals with depression (29, 30). Two commonalities among these previous two studies are especially relevant to this intervention: the use of college students and the use of those on a waitlist to receive care as the control group members. Recent research suggests that first-generation college students (FGCS) experience more anxiety and depression than non-FGCSs (31, 32). These two commonalities are relevant because they highlight a population of students who would potentially benefit from an ACT intervention and by bringing to attention the possibility of an ACT-based intervention to improving access to care.

To summarize, mental health apps clearly expand access to care; however, the actual engagement of the user is not well scrutinized, leaving the question of which components of health care apps contribute to efficacy. As an effective transdiagnostic treatment, ACT addresses the accessibility gap, but the question still remains: can individuals in need of care independently learn from an ACT-based mHealth intervention? This is especially relevant to those who have access to mobile technology, but not a health care provider. Further, could such an intervention be effective for individuals with a range of diagnoses and needs?

The present study sought to investigate engagement with and learning from an ACT-based mental health app in two cohorts:

### First-Generation College Students

FGCSs experience unique and significant distress compared to non-first-generation students. FGCSs indicate a lesser sense of belonging on average and poorer mental health on average than non-FGCSs (32); and needing but not using counseling and/or psychological services at a greater rate than non-first-generation students (32). This supports findings from the 2012 National Postsecondary Student Aid Study (NPSAS:12), a prospective study examining a sample of students who began postsecondary education in the 2011–2012 academic year (33). In 2014, follow-up data collection of over 24,000 students found that FGCSs (14%) utilized campus health services < non-FGCSs (29%) (34). First-generation status is associated with known risk factors for mental illness, such as coming from a low socioeconomic status (SES) household or belonging to a historically marginalized racial or ethnic group (35–37).

### Bipolar Disorder

BP is a chronic mood disorder characterized by dynamic episodes of depression and mania or hypomania (38). BP affects an estimated 45 million people worldwide (39), with one-third to one-half of those with BP experiencing a suicide attempt at least once in their lifetime (40). Clinical manifestations and patterns of BP are highly variable and often require a combination of medication and psychotherapy for treatment (41). However, those with BP may be more likely to have limited access to healthcare and therefore go longer before initially receiving mental health care (42). Despite the possibility of psychotherapy and medication to treat BP, clinical care often remains fragmented with a lack of clinical integration and reduced access to care (12, 42, 43). Non-adherence to medication and inconsistent access to care, including psychosocial interventions, are common obstacles leading to a worsened disease course (43).

The two-cohort model herein was used to evaluate whether the same intervention content was learned similarly by two diagnostically and demographically distinct samples. In the intervention, participants were tasked with independently learning complex emotional and psychological phenomena and applying the underlying ACT concepts to their own lives in order to make behavioral change. We predicted participants would engage with the intervention prompts to develop increased self-awareness, as observed by the content of responses. Via twice-daily assessments, we anticipated that flexibility would be observed in both behavioral form and function of the identified behaviors.

## Materials and Methods

### Cohorts

#### First-Generation College Students

First-generation college students (FGCSs) are defined in this study as students whose parent(s) or legal guardian(s) have attained less education than a bachelor's degree. Participants in this sample were recruited from the University of Wisconsin-Madison (UW) during the Fall 2019 and Spring 2020 academic terms. Recruitment methods included sending a mass email to first- and second-year undergraduate students, posting flyers on the UW campus, and brief presentations to students in UW lecture-style classes. Interested individuals completed an online eligibility screening. To be included in the study, individuals had to 1) be aged 18–19, 2) be enrolled as a freshman or sophomore undergraduate student at UW, 3) have access to a smartphone, 4) be a FGCS, and 5) endorse a subjectively high level of distress at the time of screening. Recruitment was ongoing at the time of the present analysis, and data from 25 participants were randomly selected for analysis in this study. This study has been approved by the Health Sciences Institutional Review Board at the University of Wisconsin-Madison [2019-0819] and is registered at clinicaltrials.gov (NCT04081662).

#### Bipolar Disorder

Participants with a diagnosis of either type I BP (BPI) or type II BP (BPII) were recruited from the Prechter Longitudinal Study of Bipolar Disorder (44) for a 6-week study. Recruitment began in September of 2019 and ended in August of 2020. Recruitment was ongoing at the time of the present analysis, and data from the first 24 participants who submitted app data were analyzed. Eligibility criteria included a diagnosis of BPI or BPII, consent to be contacted for future research, and access to a smartphone. This study was approved by institutional review boards at the University of Michigan (HUM126732) and the University of Wisconsin (2017-1322) and is registered at clinicaltrials.gov (NCT04098497).

### Study Design

A detailed description of the study methodology has been published (45). Here, only that which is relevant to current analyses is discussed.

Participants in each cohort completed a consent discussion with a member of the research team before reviewing and signing the informed consent document. They then completed baseline demographic and psychometric questionnaires. After doing so, they received instructions to download and use a free mobile app. Twice a day, participants were prompted to complete a brief log consisting of questions about current mood and behavior. The behavioral assessment asked participants to write what behavior they were engaged in at that moment (behavioral form) and to categorize it as either “toward” (motivated by values) or “away” (motivated by avoiding negative internal experiences) (behavioral function). Participants also had a 50% chance of receiving an ACT-based intervention prompt each time they completed the assessment log. When an ACT-based prompt was delivered, it was selected randomly from a list of 84 possible prompts, with the possibility of questions being repeated. The prompts were evenly divided across three core principles of ACT: 1) openness to internal experiences, 2) engagement with values, and 3) awareness of internal experiences. Participants responded to both the current behavior item and ACT-based prompts in a free-text format. The logging functionality allowed for participants to skip assessment items by submitting blank fields, and text responses had no minimum or maximum word limit.

### Qualitative Analysis

We performed qualitative analysis on intervention and behavioral assessment data from the FGCS (*n* = 25) and BP (*n* = 24) cohorts. Participants were randomly assigned to either the exploratory (FGCS *n* = 10, BP *n* = 9) or confirmatory (FGCS *n* = 15, BP *n* = 15) group. To be included in the analysis, participants must have downloaded the study app and responded at least once to logging prompts (showing that they knew how to respond).

The exploratory dataset was used to inductively establish a preliminary coding system. Two primary coders, co-first authors SH and AV, independently completed in-depth reviews of the exploratory data. Following the initial review, the research team met as a group to discuss themes and concepts of interest in the data. After identifying these data elements, we developed an initial coding system, which SH and AV then used to independently code the exploratory dataset. Results from both coders were compared and discussed by the research team to evaluate the quality of the coding system and further refine it. SH and AV then applied the refined coding system to 30 randomly selected intervention responses and 30 randomly selected behavior responses from the exploratory dataset, and once again compared results. The research team discussed final revisions to the coding system. In the final coding system, the behavioral qualitative data was coded across 5 categories: work-related behaviors, leisure behaviors, self-care behaviors, activity level, and social behaviors. Qualitative data for the interventions coded for response alignment, values, negative internal experiences, and contexts.

The primary coders then independently coded the confirmatory data using the finalized coding system, comparing results upon completion. Codes with discrepancies more than 1/3 of the time were removed from the analysis. Any remaining discrepancies were resolved by author TSG.

### Metrics of Engagement

To evaluate the quality of participant engagement with the intervention, the following metrics were recovered to describe each response: 1) submitted response, 2) non-blank response, 3) identification of the function of behaviors, 4) process alignment, 5) word count, and 6) qualitative content. A submitted response refers to a response to a prompt that is submitted by a person in the study app. A non-blank response refers to a submitted response that had any amount of text provided in the text field. We hypothesized that participants will submit non-blank responses to a majority of prompts. These first two metrics were calculated separately for behavioral and intervention responses.

Identification of the function of behaviors was determined for each behavioral response based on each individual's categorization of their current behavior as either moving them toward what matters (“values-based”) or away from negative internal experiences (“avoidant”). We predicted that participants would demonstrate flexibility in the function of behaviors in terms of being able to categorize the same or similar behaviors as both values-based and avoidant over the course of their intervention period (e.g., categorizing an academic behavior as values-based at one time point, and categorizing another academic behavior as avoidant at a different time point).

Process alignment was determined for each intervention response during the coding process. Each intervention prompt was designed to align with one of three core ACT processes: openness, awareness, or engagement. Responses were coded to reflect whether or not the responses were “process-aligned,” meaning that participants addressed the intended process in their response. Process-alignment was thought to indicate meaningful engagement; we hypothesized that the majority (more than 50%) of responses would be process-aligned.

Word count of a response was calculated using the function *wordCloudCounts* in Matlab (Mathworks; Natick, Massachusetts). This function splits the text into words, removes stop words, and combines words with a common root. Word counts were calculated for both behavioral assessment responses and intervention responses. We predicted that participants would respond to prompts with multiple words (non-yes/no). The final metric, qualitative content of responses, was determined using the categories established in the qualitative coding system. We examined whether or not a response fell into a certain category.

Descriptive statistics were calculated for the metrics of engagement. This was done in two steps: first, across responses per participant and then across participants. Each metric was summarized as a count, a proportion, or average across responses for each participant. For example, we calculated the total number of submitted responses and the proportion of submitted responses that are non-blank for each person; each calculated separately for behavioral and intervention responses. We then calculated information about the distribution of these participant-summarized metrics of engagement: min, max, median, and 25th and 75th percentiles. Metrics of engagement are reported in text as medians across participants to represent the majority of participants, which corresponds to the 50^th^ percentile among the tables. Majority is thus defined as over 50% of participants. To improve readability in the Results section, we will refer to a median as a value in which “over 50% of participants” had an equal or higher value. A final check was to examine whether our participant-summarized metrics of engagement were providing consistent information. To this end, we used a Pearson correlation coefficient to measure correlation between the participant-summarized metrics.

## Results

### First-Generation College Student Cohort

#### Sample Characteristics

In both the exploratory and confirmatory samples, the majority of the subjects were female (88%), comprising 9 out of 10 participants and 13 out of 15 participants, respectively. A majority of participants identified as White (50% of exploratory sample; 67% of confirmatory), and a single participant in the exploratory sample identified as Hispanic. No participants in either sample reported working full-time at the time of the study. Four (40%) participants in the exploratory group and 8 (53%) of the participants in the confirmatory group were working part-time. Two exploratory participants and 1 confirmatory participant were currently using SNAP benefits (“food stamps”), and 7 (70%) participants in the confirmatory group and 9 (60%) participants in the exploratory group reported experiencing financial problems during childhood. Data on prior history of mental health treatment or therapy was not collected. The average number of behavior responses was 68.7 (*SD* = 60.2) for the exploratory group and 54.1 (*SD* = 28.1) for the confirmatory group. The average number of intervention response was 34.4 (*SD* = 29.5) for the exploratory group and 26.2 (*SD* = 15.3) for the confirmatory group. The complete sample characteristics are summarized in Table 1.

**Table 1:** Sample characteristics by cohort.

		**Exploratory**	**Confirmatory**
		**FGCS (*n* = 10 & *n* = 15):**	
Age, mean (SD)		18.7 (0.48)	18.4 (0.51)
Gender, *N* (%)	Man	1 (10%)	1 (7%)
	Woman	9 (90%)	13 (87%)
	Unknown	0 (0%)	1 (7%)
Race, *N* (%)	Caucasian	5 (50%)	10 (67%)
	Native American	0 (0%)	0 (0%)
	African American	2 (20%)	1 (7%)
	Asian/Indian	3 (30%)	3 (20%)
	Pacific Islander	0 (0%)	0 (0%)
	More than one race	0 (0%)	1 (7%)
Ethnicity, *N* (%)	Hispanic	1 (10%)	0 (0%)
Sexual orientation, *N* (%)	Heterosexual	9 (90%)	12 (80%)
	Homosexual	1 (10%)	0 (0%)
	Bisexual	0 (0%)	2 (13%)
	Pansexual	0 (0%)	1 (7%)
	Single	5 (50%)	11 (73%)
	Partnered	5 (50%)	4 (27%)
Employment, *N* (%)	Working full time	0 (0%)	0 (0%)
	Working part-time	4 (40%)	8 (53%)
	Unemployed	6 (60%)	7 (47%)
Using SNAP benefits (“food stamps”) at time of study, *N* (%)	Yes	2 (20%)	1 (7%)
Experienced financial problems in childhood, *N* (%)	Yes	7 (70%)	9 (60%)
Children, *N* (%)	Yes	0 (0%)	0 (0%)
Behavior responses, mean (SD)		68.7 (60.2)	54.1 (28.1)
Intervention responses, mean (SD)		34.4 (29.5)	26.2 (15.3)
		**BP (*n* = 9 & *n* = 15):**	
Age, mean (SD)		41.3 (10.4)	42.0 (12.4)
Sex, *N* (%)	Female	6 (67%)	9 (60%)
	White	8 (89%)	11 (73%)
	American Indian or Alaskan Native	0 (0%)	1 (7%)
	Black or African American	1 (11%)	1 (7%)
	More than one race	0 (0%)	2 (13%)
Ethnicity, *N* (%)	Hispanic	1 (11%)	1 (7%)
Bipolar Type, *N* (%)	Type I	7 (78%)	13 (87%)
	Type II	2 (22%)	2 (13%)
Behavior responses, mean (SD)		72.2 (18.3)	67.1 (23.2)
Intervention responses, mean (SD)		33.6 (9.1)	32.6 (11.4)

#### Metrics of Engagement

**Figure 1:**
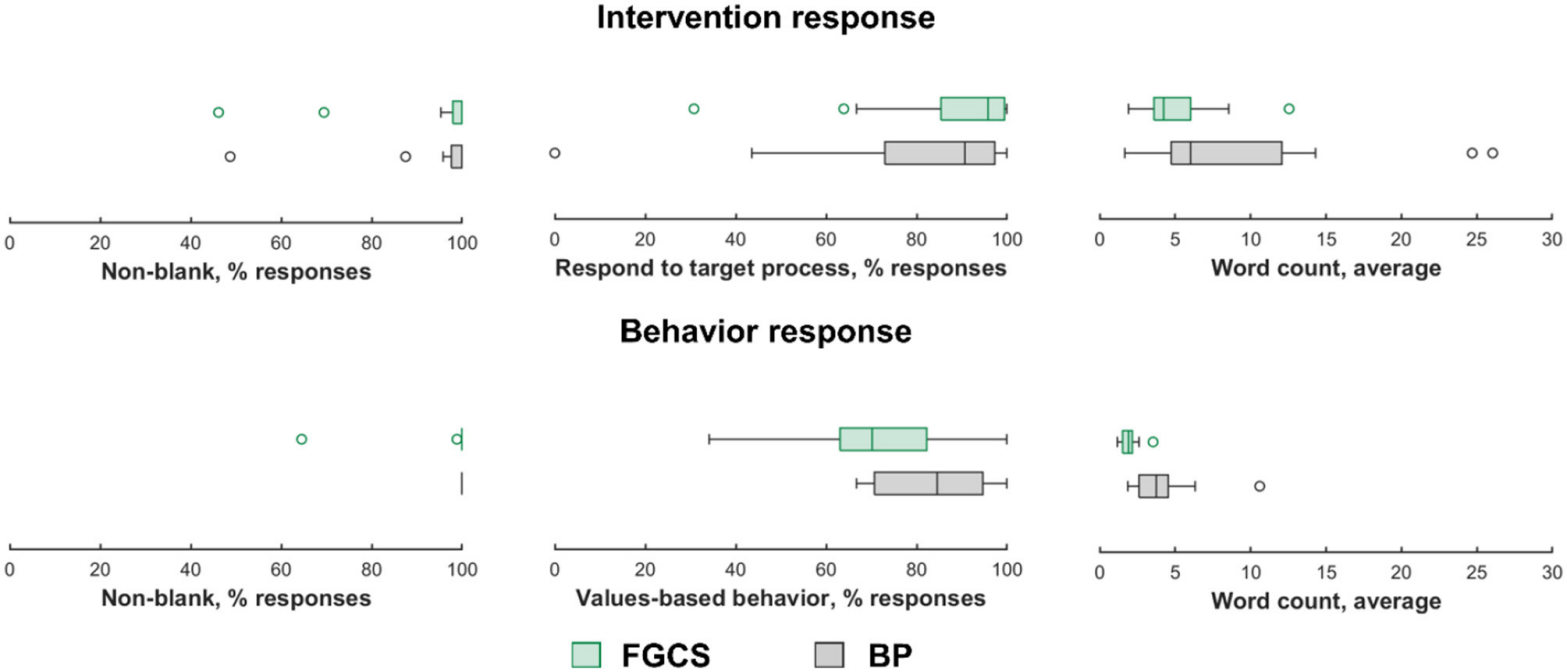
Distribution of metrics of engagement across participants.

Across all participants in the confirmatory sample, 799 behavior responses were submitted. Submitted behavior responses were accompanied by an additional ACT-based intervention prompt for a total 393 times (49.1% behavior responses coinciding with the 50-50 randomization for delivering an intervention prompt). Participants submitted a response to these prompts 372 times. Four participants accounted for all blank responses submitted (21 in total), whereas the remaining 11 participants submitted a text response to every intervention prompt received. In other words, over 50% of participants always provided a non-blank response to intervention prompts. Similarly, over 50% of participants always provided a non-blank response to behavior prompts. In addition, over 50% of participants provided responses with average word counts that were at least 4.26 words in length for intervention prompts and 1.91 words for behavior prompts. The distribution of these metrics across participants are summarized in Figure 1.

Participant-average word count for behavior responses was positively, but not significantly, correlated with participant-average word count for intervention responses (*r* = 0.44, *p* < 0.1; Table 2). In turn, these two metrics were each positively, but not significantly, correlated with percent of non-blank intervention responses (*r* = 0.31 and *r* = 0.26, respectively; *p* > 0.1). Non-blank intervention responses were also significantly correlated with non-blank behavior responses (*r* = 0.86, *p* < 0.001).

**Table 2:** Correlation between metrics of engagement with ACT processes.

		**Intervention**		**Behavior**		
		**Aligned with target process, %**	**Word count, average**	**Non-blank response, %**	**Values-based behavior, %**	**Word count, average**
		FGCS:				
Intervention	Non-blank response, %	0.90^***^	0.31	0.86^***^	0.07	0.26
	Aligned with target process, %	-	0.40	0.80^***^	0.17	0.25
	Word count, average	-	-	0.31	0.12	0.44^*^
Behavior	Non-blank response, %	-	-	-	0.06	0.34
	Values-based behavior, %	-	-	-	-	0.19
		BP:				
Intervention	Non-blank response, %	0.41	0.37	n/a^a^	0.19	0.32
	Aligned with target process, %	-	0.20	n/a^a^	−0.21	0.16
	Word count, average	-	-		−0.01	0.83^***^
Behavior	Non-blank response, %	-	-	-	n/a^a^	n/a^a^
	Values-based behavior, %	-	-	-	-	0.05

* *P < 0.1,*

*** *P < 0.001.*

a *Correlation is not defined, since all BP participants provided non-blank responses to all behavior prompts*.

##### Process Alignment

Over 50% of participants provided a collection of intervention responses in which over 96% were coded as process-aligned. Further, the percentage of process-aligned responses was significantly and positively correlated with percent non-blank intervention responses (*r* = 0.90, *p* < 0.001) and percent non-blank behavior responses (*r* = 0.80, *p* < 0.001). Percentage of process-aligned responses also correlated positively but not significantly with intervention word count (*r* = 0.40, *p* > 0.10).

##### Identifying Function of Behaviors: Values-Based or Avoidant

Behaviors, in general and those belonging to a specific behavior code, were more likely to be identified by participants as being values-based as opposed to avoidant (Table 3). Active behaviors, academic behaviors, exercise, social behaviors, and reading were the most likely to be categorized as values-based; specifically, at least half the participants always categorized these behaviors as values-based. At the 25th percentile, however, there was variation in behavioral function for each of those behavior types. Watching (e.g., TV) and sedentary behaviors were the least likely to be categorized as values-based behaviors.

**Table 3:** Among different behaviors, distribution of percent responses categorized as value-based across participants.

**Code**	**Participant percentile**					
	**Min**	**25th**	**Median**	**75th**	**Max**	***N***
	FGCS:					
Watching	0%	31%	50%	93%	100%	14
Sedentary	20%	59%	68%	78%	100%	15
Needs	46%	68%	84%	100%	100%	14
Leisure Other	0%	42%	93%	100%	100%	11
Reading	0%	55%	100%	100%	100%	9
Active	50%	80%	100%	100%	100%	15
School	50%	86%	100%	100%	100%	15
Exercise	50%	89%	100%	100%	100%	13
Social	75%	95%	100%	100%	100%	12
	BP:					
Needs	44%	77%	94%	100%	100%	15
Leisure Other	38%	86%	88%	100%	100%	15
Social	57%	85%	100%	100%	100%	15
Watching	20%	45%	69%	100%	100%	13
Work	37%	64%	95%	100%	100%	12
Media	0%	33%	56%	100%	100%	14
Reading	0%	88%	100%	100%	100%	8
Exercise	89%	100%	100%	100%	100%	11
Service	88%	98%	100%	100%	100%	8
Subjective	0%	25%	56%	70%	100%	9
Sedentary	48%	58%	81%	90%	100%	15
Active	74%	88%	100%	100%	100%	15

*Each metric was first summarized as a percentage or average across responses for each participant. We then calculated information about the distribution of these participant-summarized metrics in terms of a min, max, median, and 25th and 75th percentile, which are reported in the table above*.

##### Qualitative Themes

Over 50% of participants provided a collection of behavior responses in which at least 25% were academic related, 79% were sedentary, 10% were active, and 15% were related to self-care (Table 4). In the intervention responses, the dominant values were family relationships and education. The theme of time as a cost of engaging in values-based behaviors also emerged. The most mentioned types of negative affect were sadness and feeling overwhelmed or stressed. The concept of psychological flexibility also appeared in the intervention responses, with greater indications of flexibility than inflexibility.

**Table 4:** Distribution of percent responses with a given code across participants in FGCS cohort.

**Response**	**Metric**	**Participant percentile**				
		**Min**	**25th**	**Median**	**75th**	**Max**
Behavior	School, %	8%	16%	25%	43%	58%
	Needs, %	0%	9%	15%	25%	68%
	Watching, %	0%	8%	14%	25%	49%
	Exercise, %	0%	3%	5%	9%	18%
	Social, %	0%	2%	4%	11%	48%
	Other leisure, %	0%	1%	5%	10%	34%
	Reading, %	0%	0%	1%	5%	38%
	Sedentary, %	40%	67%	79%	86%	92%
	Active, %	4%	6%	10%	19%	29%
Intervention	Flexible, %	0%	1%	5%	7%	11%
	Inflexible, %	0%	0%	3%	8%	33%
	Workability, %	0%	0%	3%	4%	11%
	Value - Education, %	0%	0%	5%	7%	23%
	Value - Family, %	0%	0%	5%	8%	13%
	Time, %	0%	3%	7%	15%	25%
	Sadness, %	0%	0%	5%	6%	33%
	Overwhelmed, %	0%	0%	5%	8%	23%
	Low positive affect, %	0%	0%	3%	10%	13%
	Physio, %	0%	1%	3%	8%	11%
	Positive affect, %	0%	2%	4%	10%	21%
	Interpersonal context, %	0%	0%	3%	11%	22%

*Each metric was first summarized as a percentage or average across responses for each participant. We then calculated information about the distribution of these participant-summarized metrics in terms of a min, max, median, and 25th and 75th percentile, which are reported in the table above*.

### Bipolar Cohort

#### Sample Characteristics

Exploratory and confirmatory groups were similar in demographics. The exploratory group consisted of a mean age of 41.3 years (SD = 10.4), 67% females, and 89% White. The confirmatory group had a mean age of 42 years (SD = 12.4), 60% of participants were female, and 73% were White. Overall, the sample represented a high-SES population. Seventy-eight percent of the exploratory group had BPI, and 22% had BPII. Within the confirmatory group, 87% had BPI and only 13% had BPII. At baseline, the average Hamilton Rating Scale for Depression (HRSD) was 6.20 (*SD* = 5.78) and the average Young Mania Rating Scale (YMRS) was 1.83 (*SD* = 3.29). The average number of behavior responses were similar between groups (exploratory: *M* = 72.2, *SD* = 18.3; confirmatory: *M* = 67.1, *SD* = 23.2). The average number of intervention responses was 33.6 (*SD* = 9.1) for the exploratory group and 32.6 (*SD* = 11.4) for the confirmatory group. Complete sample characteristics are summarized in Table 1.

#### Metrics of Engagement

These data are intended to indicate the degree to which a person engages with ACT processes. Over half the participants provided a non-blank response to every intervention prompt, and all participants provided non-blank responses to every behavior prompt. In addition, over 50% of participants provided responses with average word counts that were at least 6.03 words in length for intervention prompts and 3.74 words for behavior prompts. Participants were largely categorizing their behavior responses as values-based as opposed to avoidant. A full description of these metrics per percentile can be found in Figure 1.

Correlations were calculated to show whether engagement metrics were related within this qualitative analysis. Table 2 summarizes these correlations. Percent non-blank response to an intervention prompt was positively, but not significantly, correlated with percent process-aligned (*r* = 0.41, *p* > 0.10) and average word count (*r* = 0.37, *p* > 0.10). Average word count for intervention responses was significantly correlated with average word count for behavior responses (*r* = 0.83, *p* < 0.001). A weaker, and sometimes negative correlation with measures of engagement was observed with the *values-based behavior* category. This aligns with expectations that *flexibility* in categorizing behaviors as values-based is a meaningful measure of engagement as opposed to *percent* values-based behavior. We will expand on this idea below.

##### Process Alignment

Over 50% of participants provided a collection of intervention responses among which at least 91% of responses were processed aligned. This is lower than what we reported above in terms of 100% of intervention responses being non-blank for over 50% of participants.

##### Identifying Function of Behaviors: Values-Based or Avoidant

Behaviors that were always indicated as values-based by most participants included active, exercise, reading, service, and social behaviors. Unsurprisingly, behaviors that were less often categorized as values-based were watching, media, and subjective behavior. However, even behaviors largely categorized as avoidant behaviors were sometimes entered as a values-based behavior, suggesting that participants were categorizing behavior based on function in the current context. For example, not every instance of “talking with friends” is considered an avoidant behavior. It is important to note that not all categories represent the same sample size as not all participants shared the same reported behaviors. Table 3 provides percentiles for behavioral codes, along with specific sample sizes.

##### Qualitative Themes

Table 5 highlights intervention responses for the median participant as they pertain to personal aspects such as family, time, and interpersonal context. For the behavior responses, the codes demonstrated the most were sedentary, needs, and active.

**Table 5:** Distribution of percent responses with a given code across participants in BP cohort.

**Response**	**Metric**	**Participant percentile**				
		**Min**	**25th**	**Median**	**75th**	**Max**
Behavior	Needs, %	11%	24%	33%	38%	70%
	Leisure Other, %	4%	12%	17%	21%	44%
	Social, %	6%	10%	17%	30%	75%
	Watching, %	0%	7%	17%	27%	32%
	Work, %	0%	2%	14%	30%	33%
	Media, %	0%	3%	4%	7%	29%
	Reading, %	0%	0%	3%	7%	18%
	Exercise, %	0%	0%	2%	7%	25%
	Service, %	0%	0%	1%	15%	36%
	Subjective, %	0%	0%	5%	11%	33%
	Sedentary, %	24%	41%	48%	59%	68%
	Active, %	8%	18%	29%	34%	44%
Intervention	Flexible, %	0%	3%	5%	10%	29%
	Inflexible, %	0%	0%	3%	5%	9%
	Workability, %	0%	0%	3%	3%	18%
	Health, %	0%	0%	3%	12%	21%
	Education, %	0%	0%	2%	3%	8%
	Work, %	0%	0%	3%	7%	23%
	Family, %	0%	3%	8%	12%	31%
	Friend, %	0%	0%	3%	7%	46%
	Other relationship, %	0%	0%	2%	7%	31%
	Self, %	0%	0%	3%	5%	17%
	Time, %	0%	6%	8%	16%	23%
	Sadness, %	0%	0%	3%	8%	16%
	Fear, %	0%	1%	5%	9%	19%
	Anger, %	0%	0%	3%	5%	6%
	Low positive affect, %	0%	0%	3%	5%	15%
	Physio, %	0%	3%	5%	7%	43%
	Positive affect, %	0%	3%	5%	9%	15%
	Work context, %	0%	0%	3%	8%	25%
	Interpersonal context, %	0%	4%	10%	18%	57%

*Each metric was first summarized as a percentage or average across responses for each participant. We then calculated information about the distribution of these participant-summarized metrics in terms of a min, max, median, and 25th and 75th percentile, which are reported in the table above*.

## Discussion

The aim of this study was to investigate the degree to which individuals engaged with and learned from mobile ACT interventions in two different cohorts, hypothesizing that in both, over 50% of participants would respond to open-ended questions in a way that aligned with ACT processes. We considered evidence of clinically meaningful engagement to be both a willingness to provide responses that offer specific, personal context and a variability in participants' self-reported behavioral function, displaying a recognition that behaviors can be flexible in function based on context. Achieving such clinically meaningful engagement—without clinical support—is important, since an ideal intervention could be utilized despite individual barriers such as availability of care, lack of access to resources (time, financial), or treatment models limited to specific diagnoses.

In both cohorts, participants demonstrated an ability to independently grasp ACT concepts and apply them, as evidenced by high proportions of process-aligned responses and flexibility in the reported behavioral function. In the BP cohort, results show process alignment in 73% responses even in the 25th percentile of participants that provided any type of response; similarly, the FGCS cohort responses were process-aligned 85% of the time at the 25th percentile. Thus, even the participants who were least process-aligned were still process-aligned in the majority of responses (over 50% of responses). The findings show that engagement in digital health is not only possible—supporting previous research (1)—but also can be achieved in a clinically significant way under the conditions described in this study.

In addition, participants were able to independently recognize and identify functions (values-based or avoidant) for the same behavior type. This is the core skill participants needed to learn to reflect psychological flexibility. For example, in the BP cohort, “watching TV” was recorded as values-based behavior 1 day, and an avoidant away behavior the following day. Similarly, “working” was frequently coded as both values-based and avoidant within one participant's responses. More to this point, there were differences in the likelihood of values-based vs. avoidant categorization for each behavior type. For example, exercise behaviors were almost always categorized as values-based but other types of behaviors had greater variability. Evidence of this skill is encouraging, as it suggests that users were able to grasp a major goal of ACT, which is to distinguish between form and function of a behavior. We conjecture that this process of engagement might mediate any symptom reduction from the intervention. This is even more encouraging considering that the study was only 6 weeks and some participants' only exposure to ACT might be a 20-min informational video created by the authors for this study.

We also utilized word count as a metric of engagement. Average word counts per response was small (<7 words) for both intervention and behavior responses. Longer average word counts were found in the BP cohort vs. FGCS cohort and for intervention vs. behavior responses. Longer intervention responses are expected given that many behaviors can be expressed concisely, whereas certain intervention prompts demanded significant reflection (e.g., “What are the consequences [positive or negative] when you try to interpret your thoughts and emotions?”). For example, short behavior responses included “homework, tv”, “talking with friends,” or “studying” as opposed to more descriptive intervention responses such as “I am very very nervous about my midterm tonight.” Although average word count was small, it was encouraging to note that some responses were lengthy, and some prompts did not require a response of any length. For example, eight intervention prompts could have been read as closed questions that could be answered with a single word, percentage value, or indicator of frequency (e.g., “Does your mind ever label you ‘bad' or ‘defective'?”).

Coinciding with engagement, a major barrier to successfully implementing digital interventions is earning the trust of participants so that they feel safe inputting information into a digital platform. Given the sensitive nature of psychotherapy and emotions more broadly, an intervention such as the one studied here would not be possible if participants were unwilling to share their responses. We suspect that we gained the trust of some participants from both cohorts based on the word count and consistent content of responses. For example, one 89-word intervention response described a particularly difficult manic episode, and a 46-word response answered the question of how they avoid uncomfortable emotions: “I usually tend to avoid uncomfortable emotions often, as I try to avoid situations that would give me these emotions. Sometimes I cannot avoid them, and inevitably end up feeling depressed.”

Most responses contained specific, personal context, displaying a willingness from participants to share their thoughts and emotions not only in an app, but with the knowledge that someone would be closely reading their responses. This allowed us to identify themes among participants' values and negative internal experiences. One of the themes in the intervention responses of both cohorts was time as a cost of or barrier to engaging in a behavior (“spending time [engaging in activity]”). The concept of time management is well-documented in research concerning college students. Effective time management has been associated with improved academic performance and reduced stress (46, 47). It has also been highlighted in a 2005 qualitative study; all participants in a small sample of 8 FGCSs noted time management as a skill important for college readiness (48). Another study found first-year college students' time management to be dynamic, changing from one semester to the next depending on their ability to meet academic goals (49). This previous work seems to establish time as an important factor in decision-making and a potential barrier or facilitator to engaging with values. Observing this theme of time in FGCS participant responses could be indicative of this importance.

The concept of time-management is less documented in research concerning BP. However, everyone is faced with the opportunity cost of spending time in one manner over another (50). Borda describes patients with BP as individuals with a broad experience of time, including the ability to be reflective, actively engaged in the moment, and thoughtful about the future (51). Examples of this reflection in our study include a preoccupation with a past event “…my [past event] was a time when I had difficulty with emotions” or optimistic thoughts about the future “…take time to relax after work.” This could suggest that the ACT intervention was able to extract a commonality among BP patients—being the importance of time in their perception of self (51). More specifically, research by Rusner et al. found that with varying mood states, the concept of and connection to time may change (52). Increased mentions of time, as seen in this intervention, might provide useful insights for providers about their patients' mood states.

Academic behaviors and the values of education and family were additional dominant themes in the FGCS cohort; family was also a dominant theme in the BP cohort. Research on FGCSs' values often examines the conflict between independent social norms at academic institutions and FGCSs' experience of interdependent social norms, which place value on family (53–56).

### Limitations

The coding process presented many challenges. Although coding was completed independently by two researchers, the process was nonetheless subjective as the researchers had to make choices on the meaning behind responses. For example, the research team had to discuss what types of behaviors to code as “needs” (i.e., attending to one's needs; self-care). One decision involved coding all responses that mentioned eating as “needs” regardless of the type of food described (e.g. “cake,” “breakfast”) thereby avoiding assumptions about what constitutes self-care for a particular individual. The behavioral coding system has its own challenge when participants listed multiple behaviors in their response. This sometimes led to responses that met for multiple codes within one broad category (“went to work, then went to class” applies to two codes in the work-related behavior category: work-related and academic); in those instances, we had to choose which code to assign. It would have been more effective to allow for multiple codes.

Coding psychiatric symptoms was particularly challenging, especially considering that the two cohorts (FGCSs and BP) are very different. For example, when participants responded with the word “upset” the researchers had to decipher what exactly was meant, i.e., did upset mean “angry” or “sad?” On that same token, a response such as “irritable” could easily be coded as anger, but in particular for the BP cohort, in terms of which mood state it was pertaining to, that remains unknown. On the flip side, a response mentioning “mania” might imply different symptomatic profiles. In other words, it was impossible to know if mania should be coded under “NEG-ANGRY” indicating the person was experiencing irritability, which is common, but not necessary, for mania. Such responses highlight the difficulty in translating these codes into their clinical significance. Future attempts at implementing ACT as a mental health app should avoid similar discrepancies by expanding the codebook to include more specific codes for what mood state a response might refer to, and potentially supplement with a form of passive data collection.

Moreover, the codes utilized with these two specific samples may not generalize to other samples, and future work might expand qualitative codes to those that are generalizable across large samples. Nevertheless, the intention of conducting parallel trials with two distinct samples was to investigate the transdiagnostic nature of the intervention and engagement with the intervention. As such, although some codes were not applicable or shifted in meaning across samples, others applied in both samples, and the similarities and differences between samples both provided important information.

The coding system is also incomplete in the sense that some responses had no codes applied to them. No codes were applied to 199 (32%) intervention responses and 8 (1%) behavior responses for the FGCS cohort, and 148 (32%) intervention responses and 11 (1%) behavior responses for the BP cohort. Several factors may have contributed: certain questions that prompted shorter responses (e.g., “yes” or “no”); no minimum word count required to submit a response; and qualitative codes removed from analysis due to low inter-coder reliability, leaving some topics unidentified, such as guilt and positive thoughts toward self. The coding system may have missed themes because of intervention prompts were too variable; intervention prompts were randomly selected from a list of 84 prompts from 3 target processes. Each target process category can be expected to elicit certain themes. Therefore, with each participant receiving a different number and assortment of prompts over their intervention period, it makes sense that high thematic frequencies were not observed within the intervention response data. By contrast, behavior responses gave us more consistent information about themes since the prompt was always the same (“What behavior are you currently engaged in?”).

It is also important to note that we compared coded categories of behavior and not distinct behaviors. For example, behaviors coded as work-related included responses such as “Planning for the class I teach and chores…,” “working,” and “writing a cover letter for a job I was invited to apply for.” Further, although it was possible for a participant's behavior response to fall under multiple behavior types (“at the movies with friends” would be 1) a watching behavior 2) a social behavior), analyses did not examine relationships between behavioral function categorization and multiple categories. Most importantly, for anyone logging a response, what the participant was actually doing at the time of response was responding to the app, and the behavior identified was presumed to be what the person was doing just prior.

Because qualitative data were collected through an app rather than in-person by a member of the research team, we were unable to clarify any response content or seek further context. A different data collection method, such as qualitative interviewing, would yield richer data than our qualitative survey items and would provide the opportunity for clarification of responses. It would also allow us to seek insight from participants on what they found to be barriers and facilitators to engagement. In light of this, findings regarding the frequency of certain themes, such as academically oriented behaviors, have been interpreted conservatively, as has been recommended by qualitative methodologists (57).

Study design factors may have influenced the frequency of engagement observed. The compensation structure for the FGCS cohort encouraged participants to continue using the app throughout the study period. Participants were compensated on a weekly basis for each week in which they responded to the app at least 50% of the time. For the BP cohort, compensation was determined based on weeks in the study, and response rate was not a factor. Another feature of the study design – specifically, the intervention itself – was accessibility. Designed for convenience, the study app allowed participants to choose when to respond. Notification functionality provided automatic reminders to log both after waking up and before bed. Each time window in which a participant could choose to respond was 5 h long, minimizing the chance that they would receive a reminder at a time when they were otherwise engaged. The brevity of the intervention meant that participants had to expend a minimal amount of time and effort to complete a log and intervention.

Variable engagement across participants may arise from some study participants having treatment experience with mindfulness-based therapy or ACT. Participants in the BP cohort were not only older than the FGCS cohort on average (42 compared to 18.7 years old, respectively), each had a psychiatric diagnosis and a history of treatment. It is possible that participants from either cohort could have been familiar with mindfulness or ACT concepts prior to participating in this study. The generalizability of our findings is also limited by small sample sizes and a lack of demographic representativeness. BP results were not representative of the general population as the majority of participants were White women, currently not identified as employed. The participants were recruited from an established longitudinal cohort that has shown a relatively high degree of trust toward the research team. Even within the BP population, our sample consisted mainly of individuals with BPI. Lastly, we are certain that the BP cohort had been diagnosed but may or may not be engaged in psychiatric treatment, and information pertaining to current medications was not collected; diagnostic and treatment history are unknown among the FGCS cohort.

Another limitation related to the mobile app concerns the 20-min introductory video that participants were instructed to watch before using the study app. The video reviewed the central concepts to be utilized throughout the assessments (form and function of behavior, personal values, internal experiences) and interventions. The video is lengthy, and it is possible that participants watched part or none of the video before using the app. Upon setting up the app for the first time, it was possible to skip the video by indicating they had already viewed it. As a result, a lack of understanding of the ACT concepts addressed by intervention prompts could have inhibited participants' abilities to engage in a meaningful way. Moreover, our inability to confirm whether the video was watched or attended to is an important limitation.

### Future Directions

Future directions with this mental health app include an evaluation of its effectiveness in promoting behavioral change and symptom reduction over time. As far as changing or improving the intervention itself, the ACT intervention prompts could be designed to elicit more person context to help limit ambiguity when coding, and a refined prompt list produced. Furthermore, if we want to elicit more consistent information between participants, the delivery of prompts could be modified: perhaps participants would benefit from an intervention in which the delivery of prompts was structured to follow a specific “lesson plan” (for example, learning about engagement with values in week 1, awareness of internal experiences in week 2, etc.). In this case, the modified study design would provide an opportunity to test a different approach to delivery and a dataset in which every participant received the same set of prompts, allowing for a more direct comparison of the content of responses. Additional information might also be collected from passive sensors, such as GPS or activity trackers, in an effort to provide more context behind individual responses. Future iterations of the intervention could also be tailored to specific study populations, expanding analysis to include diagnosis-specific outcomes.

## Conclusion

Participants from two diagnostically and demographically distinct cohorts were able to independently learn and apply complex ACT processes in the context of their own lives, as demonstrated by participants' self-reported flexibility in the form and function of behaviors. The majority of qualitative responses were specific and personal, showing that asking participants to engage with ACT in this manner can prompt reflection and meaningful engagement. ACT holds promise as the basis of a mobile intervention that can work for transdiagnostic populations.

## Data Availability Statement

The full datasets presented in this article are not readily available to maintain patient confidentiality and participant privacy. A limited dataset that includes response codes but not individual responses and that supports the conclusions of this article will be made available by the authors, without undue reservation. Requests to access the datasets should be directed to AC; cochran4@wisc.edu.

## Ethics Statement

The FGCS cohort study was approved by the Health Sciences Institutional Review Board (IRB) at the University of Wisconsin-Madison (ID 2019-0819). All participants provided electronically written informed consent. The BP cohort study was approved by University of Michigan Medical School Institutional Review Board (IRBMED, HUM# 126732). All participants provided written informed consent to participate in the study.

## Author Contributions

ET, AC, SH, and ZS designed the FGCS cohort study. ET, AC, SH, and MM designed the BP cohort study. ET wrote the ACT intervention, and AC created the mobile app. SH, TSG, ET, AC, and ZS implemented the FGCS cohort study. AV, AC, and MM implemented the BP cohort study. SM, ET, and AC designed the approach to statistical analysis. SH and AV created codebook that was reviewed and edited by TSG, AC, and ET. SH and AV coded all participant responses, and TSG acted as a third coder to resolve discrepancies. TSG wrote the methods section. SH and AV drafted the remaining manuscript with ET, AC, TSG, MM, and ZS providing edits and feedback. AC completed statistical analyses and figures. All authors approved the final manuscript prior to submission.

## Funding

The BP cohort research was supported by the Heinz C. Prechter Bipolar Research Program at the University of Michigan; the Richard Tam Foundation; and the US Department of Health and Human Services, National Institutes of Health (NIH) (K01 MH112876). Funding for the FGCS cohort study was provided by the Baldwin Wisconsin Idea Endowment, Seed Project Grant and the Clinical and Translational Science Award (CSTA) program through the NIH National Center for Advancing Translational Sciences (NCATS) (grant ULITR002373).

## Conflict of Interest

MM has consulted for Janssen and Otsuka Pharmaceuticals and received research support from Janssen in the past 5 years, all unrelated to the current work. ZS has received research and salary support from the National Institute of Health and the Center for Disease Control and has consulted for Sage Therapeutics. The remaining authors declare that the research was conducted in the absence of any commercial or financial relationships that could be construed as a potential conflict of interest.

## Publisher's Note

All claims expressed in this article are solely those of the authors and do not necessarily represent those of their affiliated organizations, or those of the publisher, the editors and the reviewers. Any product that may be evaluated in this article, or claim that may be made by its manufacturer, is not guaranteed or endorsed by the publisher.
